# KRAS and BRAF mutations in Iranian colorectal cancer patients: A systematic review and meta-analysis

**DOI:** 10.22088/cjim.11.4.355

**Published:** 2020

**Authors:** Abolfazl Yari, Asiyeh Afzali, Mostafa Aalipour, Mehran Nakheai, Mohammad Javad Zahedi

**Affiliations:** 1 *Student Research Committee, Kerman University of Medical Sciences, Kerman, Iran.*; 2 *Department of Medical Genetics, Afzalipour School of Medicine, Kerman University of Medical Sciences, Kerman, Iran*; 3 *Department of Medical Laboratory of Sciences, Iran University of Medical Sciences, Tehran, Iran *; 4 *Department of Immunology, Afzalipour School of Medicine, Kerman University of Medical Sciences, Kerman, Iran*; 5 *Department of Epidemiology and Biostatistics, School of Public Health, Kerman University of Medical Sciences, Kerman, Iran *; 6 Gastroenterology and Hepatology Research Center, Department of Internal Medicine, Kerman University of Medical Sciences, Kerman, Iran

**Keywords:** Colorectal cancer, CRC, Mutation, KRAS, BRAF, Iran

## Abstract

**Background:**

Mutations in the EGFR signaling pathway play an important role in the development of colorectal cancer (CRC). Mutations in these genes, like KRAS and BRAF, affect the treatment strategies and associated with poor prognosis and relative resistance to anti-EGFR therapies. Our aim was to conduct a systematic and meta-analysis on all studies that have been conducted on the prevalence of these gene mutations in Iranian CRC patients.

**Methods:**

Four science citation index databases (MEDLINE, EMBASE, Web of Science and Cochrane library) and local databases were searched up to March 2018 with related keywords. Two reviewers independently screened and extracted the data. Quality of all included studies was assessed using an adapted checklist from STROBE. A random-effect model was used to calculate the total prevalence of KRAS and BRAF mutations in CRC subjects by the event rate (ER). Meta-regression was utilized to explore heterogeneity causes.

**Results:**

In total, from 573 records, 23 eligible studies (2662 patients) were included for data extraction and analysis. In 18 of 23 included studies, the prevalence of KRAS mutations was 33.9% (95% CI=30.1-37.9) with I2=65.17 (p<0.001). The occurrence of KRAS mutations in codon 12 and 13 was 76.9% (95% CI = 70.4-82.3%) with I2=84.88 (p<0.001) and 23.5% (95% CI=17.9-30.3) with I2=85.85 (p<0.001), respectively. In 9 of 23 studies, the BRAF mutation rate was 3.2% (95% CI=0.003-13.6) with I2=88.61 (p<0.001).

**Conclusion:**

The prevalence of these mutations in CRC patients shows a significant difference in the different regions of Iran, which is probably due to environmental and racial factors.

Colorectal cancer (CRC) is the third leading cause of cancer deaths around the world (1). It is the second most common cancer in the Iranian population (2,3). About half of the patients with CRC develop distant metastasis (4,5). Chemotherapy is one of the best strategies for metastatic colorectal cancer (mCRC) patients and several combinations of chemotherapeutic agents are utilized to extend survival for patients with mCRC (6). The development and progression of CRC is a multi-stage process that begins with polyps, and with the accumulation of extensive genetic alterations ultimately leads to malignant and metastatic tumors. The EGFR (Epidermal growth factor receptor) signaling pathway plays a significant role in regulating cell processes including proliferation, differentiation, cell motility and apoptosis. Mutations in oncogenic genes of this pathway such as KRAS and BRAF are commonly found in CRC patients and play a significant role in the development of metastatic colorectal cancer (7–9). In recent years, with the understanding of the molecular mechanisms involved in mCRC, therapeutic strategies have been rapidly improved with the development of molecular-targeted anti-cancer drugs. Regulating the EGFR expression has become a potential target for the prevention of mCRC progression (10).

For this purpose, the anti-EGFR antibodies, such as cetuximab and panitumumab, have been used in the treatment of patients with mCRC. To predict the patient's response to these therapies requires a series of prognostic biomarkers. The KRAS gene status is a prerequisite for the response to anti-EGFR therapy (11–13). It is known that KRAS gene mutations are associated with resistance to anti-EGFR therapy in CRC patients (14–16). Several studies have shown that KRAS mutations are present in over 30% of CRC patients and most of these occur in codon 12 (14,17–20). Moreover, other genetic alterations in the EGFR signaling pathway may be associated with poor prognosis and resistance to anti-EGFR therapy (14,21,22). The BRAF gene, like KRAS, is one of the downstream EGFR oncogenic genes, and its somatic mutations activate the EGF receptor signaling in tumor cells (23). Oncogenic BRAF mutations are associated with poor prognosis and survival in mCRC patients (24). Also, some studies have shown that mutations in this gene may be associated with resistance to anti-EGFR therapy (22,25). The prevalence of BRAF mutations is an average of 10% in CRC patients (20, 26). Several studies have already investigated the prevalence of these mutations in Iran. Most of these studies were limited by region and sample size. Therefore, systematic review of all studies can lead to a more accurate picture of the prevalence of KRAS and BRAF mutations in Iranian CRC patients. Also, one previous systematic review and meta-analysis conducted by Payandeh et.al. estimated the prevalence of KRAS mutations in Iranian CRC patients using 11 studies (27). Therefore, awareness of the prevalence of KRAS and BRAF mutations in different CRC patient groups may guide strategies for treatment and testing. Because of the small sample size or non-representative sample collection, current individual studies may not be suitable for estimating the overall rate of these mutations. The mutation rate reported in previous studies varies dramatically and the reported prevalence rates are thus unsuitable for applications to other populations. Our aim in this study is to systematically review and meta-analyze all studies that examined the frequency of KRAS and BRAF mutations in different regions of Iran to obtain a more accurate estimate of KRAS and BRAF mutation rate in Iranian CRC patients.

## Methods

**Search strategy of publication:** This research is a systematic review study and was done according to PRISMA guidelines (28) and was based on a registered protocol in the PROSPERO database (CRD42016053577, available online at https://www.crd.york.ac.uk/prospero). In this study, all related papers until March 2018 indexed in science citation index databases (EMBASE, Medline, Web of Science and Cochrane library) and local databases (Irandoc and SID) were extracted and assessed. Searching strategy was performed in the English language and was done by the combination of two groups’ free-text words and Medical Subject Headings (MeSH)/EMTREE terms (group 1: colon cancer, colorectal cancer, colon tumor, colorectal tumor, rectum, metastatic colon cancer, metastatic colorectal cancer, CRC, mCRC, colon neoplasm, colorectal neoplasm, colorectal carcinoma and colon carcinoma; group 2: KRAS, K-RAS, c-KRAS, BRAF, B-RAF and c-BRAF) and "Iran". The details of our search strategy are provided in Supplementary table 1.

**Figure 1:**
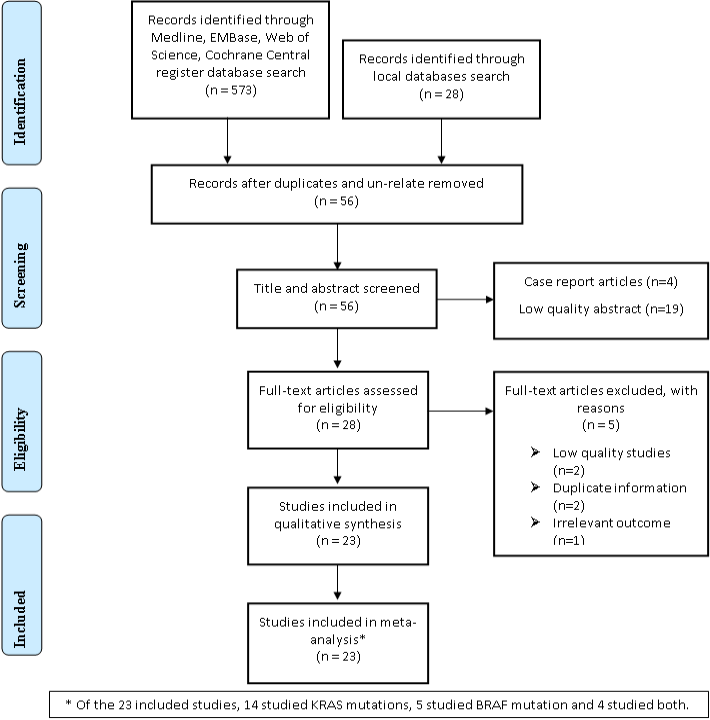
The flow chart for retrieving eligible studies used in the meta-analysis

To select the most relevant papers, searching through title, keywords and abstracts was done. The initial search included 601 articles which entered the review process for title and abstract (figure 1). Studies were selected if they met the following criteria: Cross-sectional, case series or cohort studies that evaluated the prevalence of KRAS or BRAF gene mutations in fresh frozen, FFPE or biopsy colorectal cancer samples; All studies in which the patient number with KRAS or BRAF mutations was more than one; All relevant data published by the same group or author; published data at valid international meetings; There was no limitation in methods used to detect mutations. The exclusion criteria were as follows: Studies that were unrelated to the prevalence of KRAS and BRAF mutations, such as those that focused solely on determining the clinical and pathological characteristics of the disease; Studies that examined only one of the 12 or 13 KRAS gene codons; reviews, case reports; Studies on colorectal cancer cell lines or animals, and studies that used KRAS-positive patients to monitor mutations in the BRAF gene (29).

**Quality assessment and data extraction:** Two reviewers (AY and MA) independently screened the titles and abstracts to avoid any kind of bias, and any discrepancies were resolved through discussion including a third reviewer (MJZ). The quality of studies was assessed using an adapted checklist from the STROBE (strengthening the reporting of observational studies in epidemiology) for the reporting of the quality of observational studies. Eventually, the studies that earned the minimum score entered the data extraction phase for meta-analysis. After removing unrelated, duplicated (using the Mendeley v.1.17.12 software) and low-quality articles by reviewing full papers, 23 studies (total of KRAS and BRAF) met the criteria and the following data were extracted from studies: author’s name, year of publication, location of study, sex (percentage of male), patient's mean age, sample size, clinicopathological information, mutation detection method, number of KRAS and BRAF mutations and number of KRAS mutant codons. One reviewer (AY) extracted data into Microsoft Excel 2016 sheet and second reviewer (MA) reviewed it.

**Statistical analysis:** Data analysis was performed using comprehensive meta-analysis 3.0 (CMA 3.0) software. Tests of homogeneity investigate whether the difference between studies in meta-analysis is only by chance. The degree of heterogeneity between the studies was performed using the Cochran Q heterogeneity test and the I2 statistics. A I2 value of >50% and or a Q-statistic value of p<0.05 suggests the presence of significant heterogeneity. It would be invalid to pool such data using the fixed-effects model. Meta-regression was conducted to investigate the effective factors on heterogeneity. These factors included publication year, location of study, patient's mean age, percentage of male, sample size, tumor grade, tumor stage and tumor location. Possibility of bias in reporting was checked using Begg test.

## Results

**Search results and study selection:** In total, 601 records were retrieved by searching the databases. The flowchart of search records and screening process has been summarized in figure 1. After removing the unrelated and duplicated records (n=545), the 56 remaining records were screened using the title and abstract, and the 28 records that did not meet the inclusion criteria were excluded. Then, the full-text of the remaining 28 records was reviewed in detail, of which 5 records were excluded due to the reasons given in figure 1. Finally, 23 out of 28 studies met the inclusion criteria and were included in the meta-analysis. Of the 23 included studies, 14 studies were on KRAS mutations (29, 30, 39–42, 31–38), 5 on BRAF mutations (43–47), and 4 on mutations in both (48–51).

**Figure 2:**
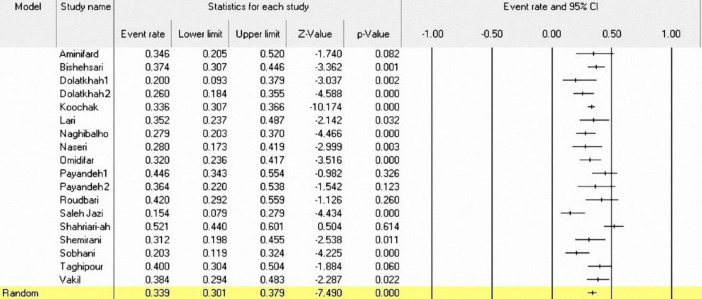
A Forest plot for the prevalence of KRAS mutation in Iranian CRC patients

**Study characteristics:** Characteristics of KRAS and BRAF mutations studies are summarized in tables 1 and 2, respectively. The 23 eligible studies included 2662 patients with a mean age of 56.26, that KRAS and BRAF mutations were screened in 2317 and 590 patients, respectively. All studies were done in cross-sectional form. Bishesari et al. (31) conducted the earliest study in September 2006; while Shahriari-Ahmadi et al. (38) conducted the latest study in June 2018. 

The sample size in the KRAS studies ranged from 33 to 1000 and in the BRAF studies from 5 to 110. Patients in ten studies were from Tehran (central) (29, 31, 32, 36, 38–40, 42, 44, 47), four studies from Shiraz (southwest) (34, 43, 45, 50), three from Kermanshah (west) (30, 35, 51), two from Tabriz (northwest) (48, 49), one study from Isfahan (central) (37), one from Yazd (central) (41), one from Ahwaz (southwest) (46) and one from Birjand (east) (33). In most studies (65.2%), FFPE samples were used and in one study, both FFPE and fresh frozen tissue were used (38). In 20 out of 23 (86.9%) studies, the incidence of CRC was higher in men than women. Different molecular methods were used for KRAS and BRAF mutations screening (tables 1 and 2); however, in most studies (60.8%), the screening method was based on polymerase chain reaction (PCR) and direct sequencing (30, 31, 47–50, 34, 37, 39, 40, 43–46). Also, the screening method was not reported in one study (41).

**Table 1:** Characteristics of the KRAS screening studies included in the meta-analysis

Author	Year	Location		Sex (%men)	Mean age (%)	Sample size (N)	Tumor Stage* 1 & 2	Tumor Stage* 3 & 4	Tumor Location* (colon)	Tumor Location* (rectum)	Tumor Grade* (poorly)	Tumor Grade* (Moderate)	Tumor Grade* (Well)	Method	Total KRAS Mutation (%)	KRAS (codon 12) %	KRAS (codon 13) %
Aminifard [117]	2016	Kermanshah		79.0	51.50	33	0.0	100	55.0	45.0	9.0	21.2	69.8	Sequencing	36.4	91.6	8.4
Bishehsari [31]	2006	Tehran		56.6	NR	182	NR	NR	71.0	29.0	NR	NR	NR	Sequencing	37.4	66.0	32.5
Dolatkhah 1[48]	2014	Tabriz		76.7	61.77	30	36.6	46.7	NR	NR	6.7	26.7	50.0	Sequencing	20.0	NR	NR
Dolatkhah 2[49]	2016	Tabriz		65.0	61.90	100	37.0	29.0	72.0	28.0	3.0	22.0	49.0	Sequencing	26.0	61.5	34.6
Koochak [29]	2016	Tehran		57.3	56.50	1000	0.0	100	NR	NR	16.4	38.4	43.9	HRMA/ Pyrosequencing	33.6	85.1	14.9
Lary [32]	2011	Tehran		59.2	54.00	54	57.4	42.6	76.0	24.0	1.9	31.5	66.6	RFLP	35.2	57.9	52.6
Naghibalhossaini [50]	2011	Shiraz		65.4	NR	110	64.5	35.5	NR	NR	43.6	40.0	4.5	RFLP/SSCP/ Sequencing	27.9	NR	NR
Naseri [33]	2016	Birjand		70.0	NR	50	NR	NR	74.0	26.0	8.0	36.0	30.0	Pyrosequencing	28.0	71.4	28.6
Omidifar [34]	2015	Shiraz		55.0	59.00	100	NR	NR	NR	NR	NR	NR	NR	Sequencing	32.0	75.0	28.2
Payandeh 1 [35]	2016	Kermanshah		61.4	57.70	83	0.0	100	61.4	38.6	7.3	32.5	60.2	HRMA/ AS-PCR/ Pyrosequencing	44.6	81.1	18.9
Payandeh 2 [51]	2015	Kermanshah		57.6	57.27	33	0.0	100	60.6	39.4	NR	NR	NR	HRMA/ AS-PCR/ Pyrosequencing	36.4	75.0	25.0
Roudbari [36]	2016	Tehran		68.0	56.96	50	NR	NR	NR	NR	NR	NR	NR	Reverse Dotbloting	42.0	71.6	28.4
Saleh Jazi [37]	2017	Esfahan		55.8	61.20	52	55.7	44.3	48.1	51.9	15.4	42.3	23.1	Sequencing	15.4	75.0	25.0
Shahriari Ahmadi [38]	2018	Tehran		54.2	52.9	144	NR	NR	79.2	20.8	18.7	64.6	16.7	RFLP/ HRMA/ Pyrosequencing	52.1	NR	NR
Shemirani [39]	2011	Tehran		76.8	49.00	48	NR	NR	NR	NR	NR	NR	NR	Sequencing	31.2	83.3	16.7
Sobhani [40]	2011	Tehran		41.4	58.00	59	NR	NR	47.5	52.5	NR	NR	NR	Sequencing	20.3	83.3	16.7
Taghipour Zahir [41]	2016	Yazd		61.2	56.00	90	47.7	52.3	76.7	23.3	2.2	38.9	35.5	NR	40.0	NR	NR
Vakil [42]	2016		Tehran	57.8	57.00	99	38.4	61.6	100	0.0	9.1	35.3	55.5	Pyrosequencing	38.4	89.4	10.5

N: Number, NR: Not reported, *: Percentage of all samples

**N: **Number, **NR: **Not reported, *: Percentage of all samples

**Table 2:** Characteristics of the BRAF screening studies included in the meta-analysis

**Author**	**Year**	**Location**	**Sex** **(%men)**	**Mean age (%)**	**Sample size (N)**	Tumor Stage* **1 & 2**	Tumor Stage* **3 & 4**	Tumor Location* (colon)	Tumor Location* (rectum)	Tumor Grade* (poorly)	Tumor Grade* (Moderate)	Tumor Grade* (Well)	**Method**	**Total BRAF Mutation** **(%)**
**Brim **[43]	2008	Shiraz	64.0	59.80	53	23.0	77.0	NR	NR	4.0	42.0	54.0	Sequencing	2.0
**Dolatkhah 1 **[48]	2014	Tabriz	76.7	61.77	30	36.6	46.7	NR	NR	6.7	26.7	50.0	Sequencing	0.0
**Dolatkhah 2 **[49]	2016	Tabriz	65.0	61.90	100	37.0	29.0	72.0	28.0	3.0	22.0	49.0	Sequencing	0.0
**Ghaffarpour **[44]	2011	Tehran	37.0	61.30	27	59.2	40.8	NR	NR	7.4	25.9	66.6	Sequencing	3.7
**Javadi **[45]	2014	Shiraz	55.0	59.80	100	66.0	34.0	100.0	0.0	0.0	17.0	83.0	Sequencing	0.0
**Naghibalhossaini **[50]	2011	Shiraz	65.4	NR	110	64.5	35.5	NR	NR	43.6	40.0	4.5	PCR-RFLP/SSCP/ Sequencing	0.0
**Mohammadi Asl **[46]	2014	Ahwaz	45.0	44.25	80	NR	NR	NR	NR	NR	NR	NR	PCR-RFLP/sequencing	46.3
**Molaie **[47]	2016	Tehran	56.4	51.00	85	NR	NR	NR	NR	NR	NR	NR	Sequencing	0.0
**Payandeh 2 **[51]	2015	Kermanshah	57.6	57.27	5	0.0	100	60.6	39.4	NR	NR	NR	HRMA/AS-PCR/ pyrosequencing	0.0

**N: **Number, **NR: **Not reported, *: Percentage of all samples

**N: **Number, **NR: **Not reported, *: Percentage of all samples

**Figure 3:**
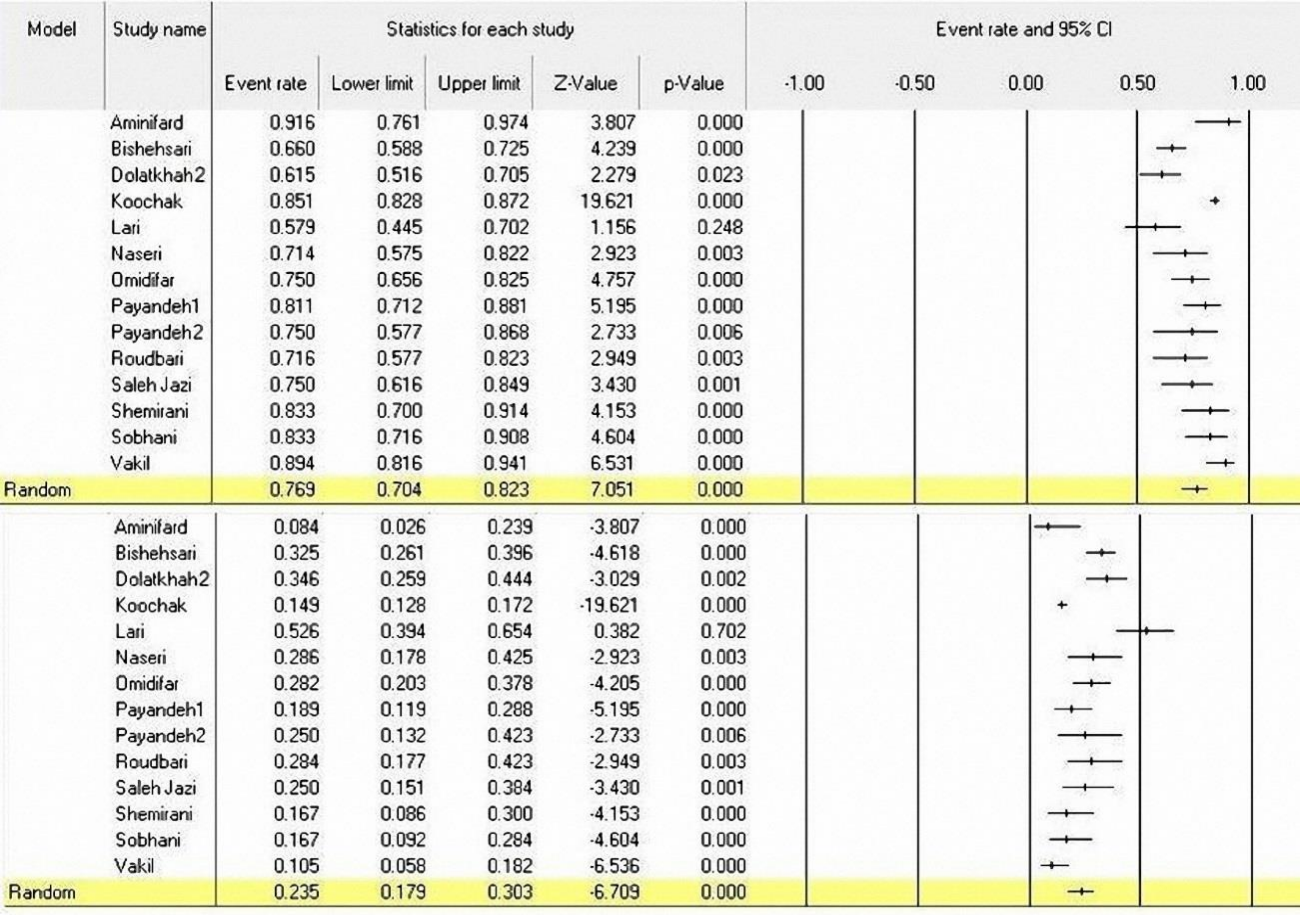
A Forest plot for the prevalence of KRAS mutant codons 12 & 13 in Iranian CRC patients

**The prevalence of KRAS mutations in CRC patients:** The prevalence of KRAS mutations has been reported in 18 of the 23 included studies (2317 patients). In these studies, the highest frequency of KRAS mutations reported by Shahriari-Ahmadi et al. was at a rate of 52.1% (95%CI: 44.0-60.1%) (38), and the lower frequency of KRAS mutations reported by Saleh Jazi et al. was at a rate of 15.4% (95%CI: 7.9-27.9%) (37). Using the random effect model, the overall prevalence of KRAS mutations was 33.9% (95%CI: 30.1-37.9%) with I2=65.17% and (p<0.001) (figure 2). Moreover, in 14 out of 18 studies, the frequency of KRAS mutations was reported in codons 12 and 13. Figure 3 shows the frequency of mutant codons among all KRAS mutations. The overall prevalence for codon 12 and 13 mutations was 76.9% (95% CI: 70.4-82.3%) with I2=84.88 (p<0.001) and 23.5% (95% CI=17.9-30.3) with I2=85.85 (p<0.001), respectively (figure 3). 

Moreover, the systematic review of included papers showed that the KRAS G12D and then KRAS G12V, were the most common amino acid change in KRAS sequence (data not shown). Given the heterogeneity of the findings of the studies and the differences in mutation rates in different regions of Iran, the possible factors causing heterogeneity were included in meta-regression analysis to find the main source of heterogeneity. The analysis showed that the year of publication, location and mean age variables were involved in heterogeneity. However, other variables such as gender, tumor grade, tumor stage, and sample size did not affect heterogeneity (table 3).

**Figure 4:**
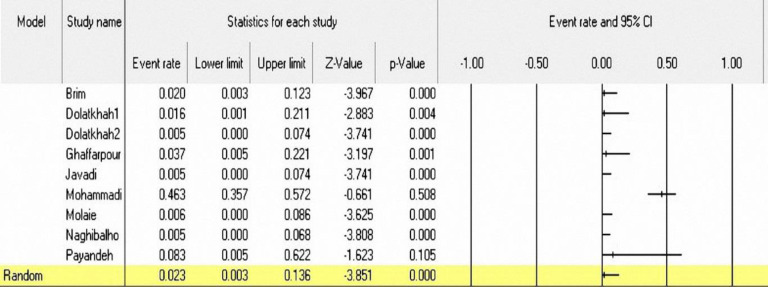
A Forest plot for the prevalence of BRAF mutation in Iranian CRC patients

**The prevalence of BRAF mutations in CRC patients:** In 9 out of 23 studies (590 patients), the prevalence of BRAF mutations in CRC patients was analyzed using the random effect model. The prevalence rates of BRAF mutations were between 0% (42,47–51) and 46.3% (95% CI: 35.7-57.2%) (46). However, the frequency of mutation in most studies (66.6%) was 0%. In more than half of the studies (5 out of 9), the screening of BRAF mutations was based on the detection of BRAF-V600E mutation. The overall prevalence of BRAF mutations was 2.3% (95% CI: 0.003-13.6%) (Figure 4) with I2=88.61 and (p<0.001). The meta-regression analysis for finding the source of heterogeneity has shown that location and means age variables are likely to be effective in heterogeneity of studies (table 4). 

**Figure 5:**
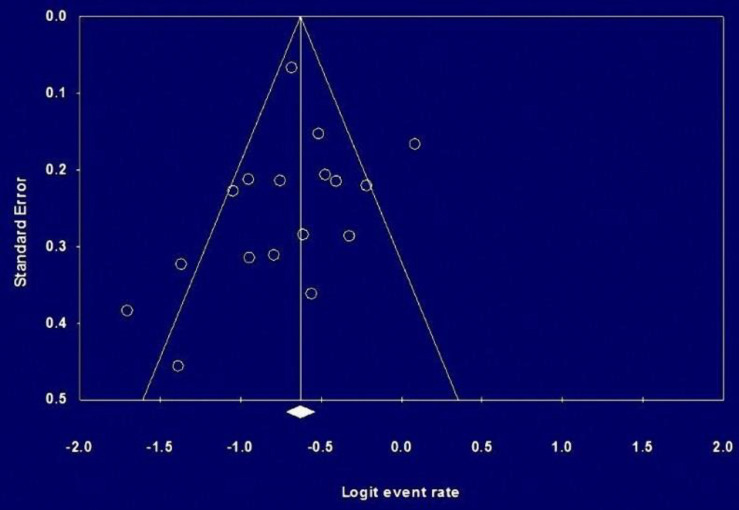
Begg Funnel plot for publication bias of the prevalence of KRAS mutation in the studies

**Figure 6:**
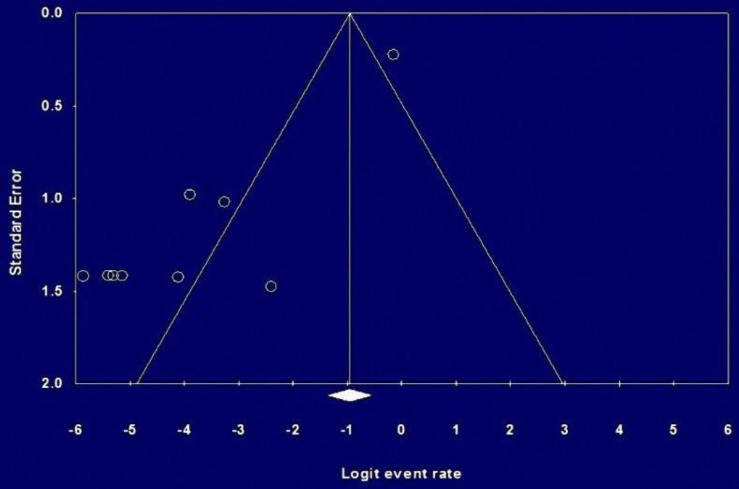
Begg Funnel plot for publication bias of the prevalence of BRAF mutation in the studies

**Publication bias:** Begg funnel plot was performed to assess the reliability of the results. To investigate the presence of publication bias, a funnel plot of random effects calculated from individual studies examined the prevalence of KRAS (figure 5) and BRAF (figure 6) mutations in Iranian CRC patients. There was no strong indication of publication bias among the studies included in the meta-analysis for KRAS mutation, but there were some biases among BRAF mutation studies.

**Table 3:** Effects of possible factors in the between-study heterogeneity in the prevalence of KRAF mutations (meta-regression model).

**Factors suspected of developing heterogeneity**		**Coefficient**	**Standard error**	**P-value**
Year	2011 2014 2015 2016 2017 2018	-0.40 -0.87 -0.18 -0.08 -1.19 -0.60	0.25 0.51 0.28 0.21 0.45 0.29	0.11 0.09 0.53 0.70 0.008* 0.03*
Location	Birjand Kermanshah Shiraz Tabriz Tehran Yazd	0.76 1.30 0.85 0.56 1.17 1.30	0.61 0.51 0.51 0.53 0.47 0.56	0.21 0.01* 0.09 0.30 0.01* 0.02*
Tumor grade	Poorly Moderately Well	-1.29 0.52 0.26	1.74 0.46 0.64	0.46 0.25 0.68
Tumor stage	1 & 2 3 & 4	-0.62 0.68	0.43 0.36	0.14 0.06
Tumor location	Colon Rectum	1.67 -1.67	0.90 0.90	0.07 0.07
Sex (male)		-0.29	1.11	0.79
Mean age		-0.07	0.03	0.01*
Sample size		0.0001	0.0004	0.81

**Table 4:** Effects of possible factors in the between-study heterogeneity in the prevalence of BRAF mutations (meta-regression model)

**Factors suspected of developing heterogeneity**		**Coefficient**	**Standard error**	**P-value**
Year	2011 2014 2015 2016	-0.36 1.01 1.49 -1.33	3.20 2.99 3.81 3.24	0.91 0.73 0.69 0.68
Location	Kermanshah Shiraz Tabriz Tehran	-2.25 -4.45 -4.56 -3.75	1.49 0.73 1.03 0.86	0.13 <0.001* <0.001* <0.001*
Tumor grade	Poorly Moderately Well	-2.24 1.69 1.51	3.82 5.45 2.35	0.55 0.75 0.52
Tumor stage	1 & 2 3 & 4	-2.41 2.89	2.22 2.04	0.28 0.16
Tumor location	Colon Rectum	- -	- -	- -
Sex (male)		-8.43	6.82	0.21
Mean age		-0.22	0.06	<0.001*
Sample size		-0.02	0.03	0.50

## Discussion

The EGFR signaling pathway plays an important role in the development of colorectal cancer. The EGF receptor is responsible for the activation of genes involved in the RAS-RAF-MAPK and PI3K-AKT pathways (23,52). Nowadays, there is a significant improvement in the treatment of mCRC using specific monoclonal antibodies (mAbs) targeting the EGF receptor (53). However, it has been found that a significant number of patients with mCRC due to the activation of the EGFR signaling pathway by the mutation in the downstream oncogene genes of this pathway (KRAS, BRAF, NRAS and PIK3CA) was associated with a poor response to this treatment (7,54,55). Screening of the oncogenic mutations in the EGFR signaling pathway is an important part of determining the therapeutic strategy for the CRC patients and their mutation status may influence their response to anti- EGFR therapy (54).

Several meta-analyses have been performed to study KRAS and BRAF mutations in melanoma (56), non-small cell lung cancer (NSCLC) (57), colorectal cancer (58,59) and papillary thyroid cancer (60). In the present study, 23 studies, including 2662 CRC patients from different regions of Iran were analyzed for the prevalence of KRAS and BRAF somatic mutations. The rate of KRAS mutations was 33.9% (95% CI: 30.1-38.0%), which is close to published data from the United States (35.7% and 35%) (61,62) (31%) (63), China (32%) (64), Japan (33.5%) (65), Taiwan (33.5%) (66), Russia (35.9%) (67), France (33.8) (68), United Kingdom (36.9%) (69) and Brazil (36%) (70); although it differs from some published data from Germany (41%) (71), Italy (62.2%, 43%, 43% and 52.2%) (72–75), Turkey (44%) (76), India (20.5% and 23%) (77,78), Pakistan (13%) (79), Saudi Arabia (42.2% and 56%) (80,81), Morocco (24%) (82), Egypt (11% and 18.4%) (83,84), Thailand (23%) (85) and Korea (20.7%) (86). The prevalence of KRAS mutations in patients with CRC varies worldwide (11-66.1%) (59,61,81–83,85–91,63,92–101,64,102–104,66,68,75–77,80). The variability of the findings of studies may be related to ethnicity, geographical area and even lifestyle (10,71,94,105,106). Given the relatively high prevalence of KRAS mutations in the Iranian CRC patients, these findings alert physicians to patients that may be at elevated risk for carrying a tumor with KRAS mutation as the focus for screening.

Based on our results, most KRAS mutations occurred in codon 12 and then codon 13. These results are approximately similar to those of other studies (17,63,76,79,82). For example, in a study conducted in Belgium, the prevalence of KRAS mutations was 36.3%, with 91% of mutations in codons 12 and 13 (107). In the study of Dobre et al., the occurrence of KRAS mutations in codons 12 and 13 was 79.3% and 19.7%, respectively (108). An Indian study reported that KRAS mutations occur in 87% and 13% of cases in codons 12 and 13, respectively (109). This is also consistent with the reported data in a large series of 989 patients from Brazil where 87% of mutations were in codon 12 and 13% in codon 13 (70). However, a study in the Greek population reported a low frequency of KRAS mutations in codon 12 (29.3%) (110). Also, our findings showed that the KRAS G12D and then KRAS G12V were the most common amino acid change (data not shown),that were consistent with published data (86,100,103,104,108).

Iran is one of the largest world's country both by geographical area and by multi-ethnic populations and this can explain the diversity of the results of various studies. The meta-regression analysis of studies also identified the location as one of the factors contributing to the heterogeneity. Although, differences in the mutation status of KRAS in different regions of Iran due to the small sample size and the absence of comprehensive studies were observed, there is no definitive result of the geographical distribution effect on the prevalence of KRAS mutations. Therefore, more comprehensive researches are necessary to further describe the mechanisms involved. For example, a large-scale study from mCRC patients reported no significant difference in the mutant codons according to geographical areas (111). These findings suggest that the location may not have a great impact on the mutant codons. In addition, the heterogeneity of the data can be explained by the difference in the data collection method, the aim of data collection and the different time periods of sample collection.

BRAF is a part of the RAF gene family that plays a role like the KRAS in the EGFR pathway. BRAF mutations are less common compared to KRAS mutations. The BRAFV600E mutation is one of the most commonly genetic changes in colorectal cancer. The distribution of BRAF mutations significantly varies from 1. 1% to 25% across the globe (26, 59, 85–91, 93–95, 61, 96–101, 103, 104, 109, 112, 64, 66, 68, 71, 75, 76, 82). The most common BRAF mutation in this study was V600E, which occurs at c.1799T>A due to substitution. This mutation results in a constitutive activation of the MAPK pathway, which modulates the cell growth signals to transcriptional activity of regulatory genes in cell cycle (113). In the present study, the prevalence of BRAFV600E mutations in Iranian CRC subjects was 3.2% (95% CI: 0.003-13.6), which is consistent with the previous findings of Asian studies (1.1% to 4.9%) (66,86,89,104,114) but lower than several Western studies (9.5% to 15.8%) (61,68,87,103,112). Heterogeneity is a problem that may influence the interpretation of the findings of meta-analyses. This study showed a high degree of heterogeneity which is why a random effect model was utilized for analysis. The meta-regression analysis showed that study location and mean age are likely to contribute to the high levels of heterogeneity. Similarly to other studies, these differences in the frequency of mutations may be influenced by race, geographic distribution, life style, and other variables of study such as mean age (115,116). The geographic region can have a great impact on the tumor mutation patterns. In this meta-analysis, nine studies that had been done in different regions of Iran, were selected for geographical optimization.

This study offers several strengths. A key strength of our study is the large number and range of studies included for estimating the overall rate of KRAS and BRAF mutations in Iranian CRC patient groups. We carried out a systematic search strategy with suitable inclusion criteria, result in the large number of studies in such a meta-analysis conducted in Iranian CRC patients. These results can act as the reference point for future researches or treatment strategies. We utilized a suitable approach to choosing the random effect model for pooling studies by taking into account the presence or absence of heterogeneity. Furthermore, we utilized tests for publication bias assessing the reliability of the results.

There were several limitations in this study that should be considered in interpreting the findings. First, one of the most important limitations of this study was the form of data reporting incomplete data, and lack of response from authors. Although all 23 studies obtained the qualitative criteria for inclusion in the analysis, even in these articles, as shown in tables 1 and 2, some important characteristics such as patients mean age, distant metastasis, differentiation and tumor stage are not mentioned. Second, a search of the gray literature was not conducted, and therefore publication bias could not be removed entirely. Third, in many studies, several hotspot regions such as the exon 4 of the KRAS gene and exon 11 of the BRAF gene were not screened. Fourth, some studies reported the mutation prevalence only in mCRC patients. Fifth, the number of studies on BRAF mutations was low and in most studies, mutation screening conducted just for the BRAFV600E resulting in the possibility of bias. Also, the small sample size of published studies increased the risk of bias. Therefore, further studies on BRAF mutations seem necessary to prove our study results. Sixth, heterogeneity between studies like the study design and methods for diagnosis of KRAS and BRAF mutations may potentially affect the study results. Finally, we did not investigate the relationship between mutations and the clinical stage in many studies due to lack of data.

In conclusion despite some limitations, we achieved remarkable results in this meta-analysis. This meta-analysis showed that the total prevalence of KRAS and BRAF mutations varies in different regions of Iran. Moreover, comparing the results of this study with other studies showed that the prevalence of these mutations in Iranian patients with CRC is also diverse in comparison with other populations, which may be related to ethnicity, geographical distribution and lifestyle. 

To targeted treatment process of CRC patients, reduces undesirable effects of treatment and prevents waste of resources, estimating an accurate overall rate of KRAS and BRAF mutations in Iranian patients is important. Finally, this study recommends that due to high rate of KRAS mutations in Iranian patients with CRC, it should be considered in determining treatment strategies.

## Authors’ contributions

AY contributed to the concept and design of the study. AY and MA designed and carried out search strategies. AA and MN conducted the meta-analysis and interpretation of data. AY, AA and MA drafted the manuscript. MJZ supervised the findings of this work and reviewed the final manuscript. All authors read, revised and confirmed the manuscript.
